# Boosting the In Vivo Transdermal Bioavailability of Asenapine Maleate Using Novel Lavender Oil-Based Lipid Nanocapsules for Management of Schizophrenia

**DOI:** 10.3390/pharmaceutics15020490

**Published:** 2023-02-01

**Authors:** Fatma Sa’eed El-Tokhy, Mona M. A. Abdel-Mottaleb, Sherif S. Abdel Mageed, Abdulla M. A. Mahmoud, Elsayed A. El-Ghany, Ahmed S. Geneidi

**Affiliations:** 1 Department of Pharmaceutics, Faculty of Pharmacy, Badr University in Cairo (BUC), Badr City 11829, Egypt; 2 Department of Pharmaceutics and Industrial Pharmacy, Faculty of Pharmacy, Ain Shams University, Cairo 11566, Egypt; 3 Pharmacology and Toxicology Department, Faculty of Pharmacy, Badr University in Cairo, Badr City 11829, Egypt

**Keywords:** asenapine maleate, lipid nanocapsules, medium-chain triglycerides, lavender oil, transdermal drug delivery

## Abstract

Lipid nanocapsules (LNCs) are promising for transdermal drug delivery due to their higher permeability-enhancing effects compared to polymeric nanoparticles. Lavender oil is an essential oil consisting of several terpenes (primarily linalool and linalyl acetate) known for their profound permeation-enhancing action. In the present work, we successfully encapsulated asenapine maleate (a second-generation antipsychotic that is highly metabolized by the liver, reducing its oral bioavailability) into biocompatible LNCs for transdermal application using a novel oily phase, i.e., lavender oil (LO-LNCs). A comparative study was conducted to determine the effects of different oily phases (i.e., Miglyol^®^ 812, Labrafil^®^ M1944CS, and Labrafac™ PG) on the LNCs. Surfactant types (Kolliphor^®^ HS15, Kolliphor^®^ EL and Tween80) and oil:surfactant ratios were studied. Blank and asenapine-loaded LNCs were optimized for particle size, polydispersity index, zeta potential, drug content and ex vivo skin permeation. Lavender oil and Labrafil^®^ showed smaller vesicular sizes, while LO-LNCs increased the permeation of ASP across rat skin. In vivo pharmacokinetics revealed that LO-LNCs could increase the ASP C_max_ via transdermal application by fourfold compared to oral suspension. They increased the bioavailability of ASP by up to 52% and provided sustained release for three days. The pharmacokinetic profile of the LO-LNCs was compared to ASP-loaded invasomes (discussed in a previous study) to emphasize LNCs’ transdermal delivery behavior.

## 1. Introduction

**Figure 1:**
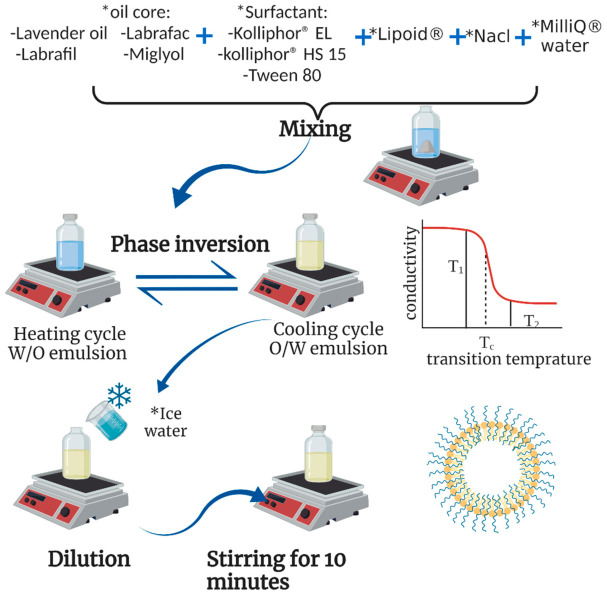
Formulation steps of lipid nanocapsules. * Essential components.

LNCs are smart blended nanocarriers, as they comprise the properties of polymeric nanoparticles and liposomes [1]. They are composed of non-toxic biocompatible materials and are prepared by a simple, low-energy, solvent-free phase-inversion process (Figure 1) that is suitable for scaling up without the use of heavy equipment. Due to their narrow particle size distribution and steric stabilization with PEGylated surfactants, their physical stability is ensured [2].

LNC formulations are mainly composed of three ingredients that are approved by the FDA for oral, topical and parenteral administration: an oily phase, an aqueous phase, and a nonionic surfactant. The oily phase is commonly a medium-chain triglyceride (MCT) with 8 to 12 carbon atoms, such as Labrafac™ PG. The second fundamental component is a hydrophilic PEGylated surfactant such as Kolliphor^®^ HS15 (formerly Solutol^®^ HS15), derived from polyethylene glycol (PEG) which decreases the interfacial tension and stabilizes the particles, thereby producing smaller particles. In addition, a small amount of a lipophilic surfactant such as lecithin is commonly used. Lecithin was found to confer hardness to the outer shell and increase the stability of the lipid nanocapsules. Moreover, it is necessary if the size range of 50–100 nm is desired. Finally, the aqueous phase consists of purified water containing sodium chloride [3,4,5]. A variety of drugs can be efficiently delivered using LNCs, including cancer therapeutics [6,7], NSAIDs [8], macromolecules such as siRNA and DNA [9], and radiotherapeutics [10]. Moreover, their oily nature improves the uptake and lymphatic absorption of poorly soluble drugs and, hence, improves their bioavailability [11].

Essential oils have been recently introduced as potential oily phases for the preparation of LNCs. In addition to acting as vehicles for drugs, their biological activities—such as antifungal, sedative, or penetration-enhancing properties—could enhance the overall clinical outcomes of LNCs. Essential oils with antifungal activity, such as eucalyptus oil and orange oil, were evaluated for their utility to prepare LNCs, as an alternative to medium-chain triglycerides [12]. Essential oils and their constituents could enhance the penetration of various drugs through topical delivery into the lower skin layers using different mechanisms of action based on disruption of the tight intercellular lipid structure between corneocytes in the stratum corneum, interacting with the intercellular domain of the protein, which induces their conformational modification or increases the drug’s penetration through skin layers [13]. Lavender oil is one of the most valuable essential oils, owing to its diverse applications as an antibacterial, antifungal, carminative (smooth-muscle-relaxing), sedative, antidepressant, and an effective treatment for burns and insect bites. Today, lavender oil is used in aromatherapy and massage. The activity of lavender oil is explained by its main components: linalool (30.6%), linalyl acetate (14.2%), geraniol (5.3%), lavandulol (4.7%), and lavandulyl acetate (4.4%). Pure lavender oil extracted from *Lavandula officinalis* was used in this work to prepare LNCs, and its effect on increasing drug permeation was evaluated.

Transdermal drug delivery (TDD) is an interesting research field due to its numerous advantages. TDD alleviates the possible reactions due to oral administration, ranging from gastrointestinal disturbances up to first-pass metabolism that could diminish the activity of various drugs. It could offer sustained release kinetics—especially for drugs with a short half-life—which, in turn, could reduce their dosing frequency. TDD is a drug delivery option that is characterized by patient acceptance and ease of application, leading to enhanced patient compliance, especially with chronic conditions such as schizophrenia [14].

Our drug model is asenapine maleate (ASP)—an atypical antipsychotic with diminished oral bioavailability (about 2%) due to first-pass metabolism. 

Therefore, the current paper aims to assess the feasibility of preparing LNCs loaded with asenapine maleate (ASP) by adopting the phase-inversion method using various types of oily phases (introducing lavender oil as a novel oily phase with natural origin) and surfactants to compare the effects of each component on the physicochemical and permeation properties of the prepared formulations, so as to enhance ASP’s bioavailability. Afterward, the best formulation was chosen as a transdermal delivery system during a subsequent in vivo experiment.

## 2. Materials and Methods

### 2.1. Materials

Asenapine maleate (ASP) was gifted by Hikma Pharmaceuticals, Giza, Egypt. Kolliphor^®^HS15 and Kolliphor^®^ EL were kindly provided by BASF Ludwigshafen, Germany. Miglyol^®^ 812 (medium-chain triglyceride; MCT) was kindly provided by Fagron GmbH, Barsbüttel, Germany. Labrafil^®^ M1944CS and Labrafac™ PG were kindly gifted by Gattefossé S.A. (Saint-Priest, France). Phospholipon^®^ 90G (soybean lecithin) was kindly gifted by Lipoid GmbH (Ludwigshafen, Germany). Lavender oil (100% pure essential oil, European Pharmacopeia) was purchased from Holland and Barrett, Nuneaton, UK. Sodium chloride, Tween 80, dipotassium hydrogen phosphate, and potassium dihydrogen phosphate were purchased from ADWIA, El-Nasr Pharmaceutical Co., Cairo, Egypt. 

### 2.2. Preparation of Lipid Nanocapsules

LNCs were prepared by the phase-inversion method followed by a sudden shock to the system using cold water at 0 °C [1]. Briefly, weighed amounts of hydrophilic surfactant (Kolliphor^®^ HS15, Kolliphor^®^ EL, or Tween 80), lipophilic surfactant (phospholipon^®^ 90G), oily phase (Labrafil^®^ M1944CS, Labrafac™ PG, lavender oil, or Miglyol^®^ 812), NaCl, and deionized water (18% *w*/*w*) were mixed using a heated magnetic stirrer. Heating continued up to a temperature of 85 °C (above PIT) until a phase inversion to a W/O emulsion was observed. The mixture was then cooled down to 60 °C, where O/W emulsion was restored. The previous heating/cooling cycle was then repeated three times before sudden cooling and dilution were performed using an equal volume of cold water at 0–4 °C. The obtained LNCs were kept under magnetic stirring for 10 min before any further characterization [15]. For the preparation of a final volume of 5 mL of ASP-loaded LNCs, 25 mg of the drug was dissolved in 50 µL of methanol and mixed with the oil phase before proceeding according to the previously described procedure. Visual examination and light microscopy were used to confirm the absence of any drug precipitates or crystals in the obtained LNCs.

Different compositions of LNCs were prepared to study the effects of three variable factors—oil type, surfactant type, and oil:surfactant ratio—on the physical properties of the obtained LNCs. The percentages of the oily phase and hydrophilic surfactant in the total formulation varied according to the oil:surfactant ratio. The studied oil:surfactant ratios were 1:1 with corresponding oily phase and surfactant percentages of 15% *w*/*v* and 15% *w*/*v*, 2:1 with 20% *w*/*v* and 10% *w*/*v*, respectively, and 5: 1 with 25% *w*/*v* and 5% *w*/*v*, respectively.

Phospholipon^®^ 90G and NaCl were kept constant in all formulations at concentrations of 1% *w*/*w*, as previously reported [12,15]. The detailed compositions of all blank LNCs in this study are described in Table 1.

### 2.3. Measurement of Particle Size and Polydispersity Index (PDI)

By using photon correlation spectroscopy, the LNCs’ particle size and size distribution were examined in terms of their average particle diameters and polydispersity index [15]. Before the analysis, LNCs were diluted 10-fold with deionized water. A Malvern Zetasizer (Worcestershire, UK) was used to perform each measurement in triplicate at 25 ± 0.5 °C and 90° to the incident beam.

### 2.4. Determination of Zeta Potential

Utilizing a laser Doppler anemometer (Worcestershire, UK) in conjunction with the Zetasizer Nano, the zeta potential of the produced LNCs was ascertained using electrophoretic light scattering technology at 25 ± 0.5 °C; the measurements were conducted in triplicate [16].

### 2.5. Determination of Drug Content in Asenapine-Maleate-Loaded LNCs

The total drug content was determined after lysis of the prepared lipid nanocapsules with absolute methanol and sonication for 10 min [17]. The samples were filtered through a 0.22 µm syringe filter, and the concentration of asenapine was determined in absolute methanol using HPLC analysis. A Waters Alliance (Milford, MA, USA) 2695 separation module outfitted with a Hibar C18 column was used to analyze the ASP using reversed-phase HPLC. The mobile phase was a filtered and degassed solution of 50:50 acetonitrile and 0.05 M potassium dihydrogen phosphate buffer solution (pH 2.7), with a flow rate of 1 mL/min, and a UV detector operating at 270 nm was used to detect the ASP. The drug was retained for 4.5 min at 25 °C, and the injection volume was 20 μL of solution [18].

### 2.6. Ex Vivo Skin Permeation and Deposition Study of ASP-Loaded LNCs

Ex vivo drug permeation from ASP-loaded LNCs across the skin was evaluated using modified Franz diffusion cells. Adult female Wistar rats weighing 180–220 g were used to excise the skin for the permeation studies. The rats were euthanatized by excessive ether anesthesia, and the hair was shaved from the dorsal side. The underlying adipose and subcutaneous tissues were carefully separated from the excised skin using a scalpel. The skin was washed and wrapped in aluminum foil and then kept frozen at −80 °C until further use. The skin was soaked in phosphate buffer (pH 7.4) for 30 min before the experiment. The skin was then mounted between the two compartments of the Franz cell, with its stratum corneum (SC) side towards the donor compartment. The receptor compartments were filled with receptor medium composed of pH 7.4 phosphate buffer containing 2% Tween 80. The effective area of diffusion was 1.5 cm^2^, and the volume of the receptor compartment was 40 mL. Aliquots of the LNCs equivalent to 10 mg of the drug were added to the donor compartments. The temperature of the receptor compartments was maintained at 37 ± 0.5 °C and stirred at 100 rpm throughout the experiment. At periodic intervals of 0.5, 1, 2, 4, 6 and 24 h, 1 mL samples of the receptor medium were collected. After each sampling interval, equal volumes of fresh release medium were replaced into the receptor compartments. The samples were then analyzed for their asenapine concentration using RP-HPLC [19,20]. The cumulative amount of drug permeated through the skin was plotted as a function of time for each formulation. Skin permeation parameters, such as transdermal flux and cumulative percentage of ASP permeated through a unit area of skin, were calculated from the plot of cumulative drug permeated versus time.

At the end of the experiment, the skin was removed from the Franz diffusion cell and then wiped using paper tissue to remove the remaining formulation on the surface. The SC layer was separated from the skin using the tape-stripping method [21]. Briefly, the SC was stripped 15 times continuously using transparent Scotch tape. Adhesive tapes were immersed in glass vials filled with 10 mL of methanol and shaken for 24 h. Then, the vial was sonicated for 15 min. The collected methanol was centrifuged at 10,000 rpm and 25 °C for 15 min and then filtered through 0.22 μm nylon syringe filters. Then the remaining layers—the epidermis and dermis—were streaked with a knife, exposing the deeper tissues to facilitate drug extraction. The streaked skin was then extracted in the same way as described for the SC. The asenapine concentrations in the extracts were then analyzed using RP-HPLC [22].

### 2.7. Evaluation of the Physical Stability of the Selected Asenapine-Maleate-Loaded LNCs upon Storage

The selected ASP-loaded LNC formulations were sealed in glass vials and stored in a refrigerator at 4 °C. The stability of the stored formulations was assessed by re-measuring the particle size, PDI and zeta potential after storage for two and six months.

### 2.8. Microscopic Examination of the Selected Asenapine-Maleate-Loaded LNCs Using High-Resolution Transmission Electron Microscopy (HR-TEM)

To study the morphology of the selected nanocapsules, a transmission electron microscopy (TEM) investigation was performed. A drop of the sample was placed on a copper grid, and any extra liquid was drained using filter paper. Then, 1% uranyl acetate dye was used to stain the LNCs and then dried. The grid was subsequently scanned using a TEM-100S instrument (Joel, Tokyo, Japan) [12].

### 2.9. Fourier-Transform Infrared (FTIR) Spectroscopy

Infrared spectroscopy was performed on the individual components and the final selected formulation. Asenapine maleate, lavender oil, Phospholipon 90G, Kolliphor^®^ HS-15, and selected blank and asenapine-loaded LNCs were scanned over a wavenumber range of 3500–1000 cm^−1^ using an FTIR Spectrophotometer (ALPHA-T, Bruker, Billerica, MA, USA) [23].

### 2.10. Bioavailability Study on the Selected LNC Formulations

#### 2.10.1. Animals

The study protocol received the approval of the Institutional Animal and Ethics Committee of the Faculty of Pharmacy at Badr University in Cairo. As a means of adaptation, for one week, 36 male Wistar rats weighing 230–250 g were given a regular diet and kept in polypropylene cages with unrestricted access to food and water. All of the animals were housed in a controlled environment of 25 ± 2 °C temperature, 60–65% relative humidity, and a 12 h light and dark cycle. The rats were divided randomly into 5 groups. The control group A comprised six healthy rats that did not receive any medication. Group B, which included six rats, received asenapine maleate solution intravenously at a dose of 1 mg/kg [24]. Group C included six rats administered orally with 15 mg/kg of ASP aqueous suspension. ASP-loaded lipid nanocapsules (F22-AS) were administered orally to the six rats in group D, and also applied topically to group E, which included eighteen rats [25].

Blood samples (250 L) were collected from rats’ retro-orbital sinus at predefined intervals (0.5, 1, 2, 3, 4, 5, 6, 24, 48 and 72 h) following administration. All rats’ blood was centrifuged for 15 min at 10,000 rpm and 4 °C to separate plasma. The plasma was kept at a temperature of −80 °C until examination. Each thawed sample was extracted with 1 mL of methanol and 2 min of vortexing. After centrifuging the mixture at 15,000 rpm for 15 min at 4 °C, the organic supernatant was recovered and filtered through a 0.22 µm nylon filter. The supernatant was evaporated and accurately reconstituted with 250 L of methanol. RP-HPLC was used for the quantitative determination of ASP concentration in each sample.

#### 2.10.2. Pharmacokinetic Analysis

PK Solver (Version 2.0) was used to evaluate the ASP plasma concentration versus time data in individual rats. A difference was regarded as statistically significant if it was less than 0.05 at the probability level. The pharmacokinetic parameters—including mean residence time (MRT), area under the curve (AUC) from zero to last time (AUC_0–t_), and area under the curve (AUC) from zero to infinity (AUC_0–∞_)—were estimated. The following equation was used to determine the absolute bioavailability of asenapine after extravenous administration [26]:

$$F_{\mathrm{absolute}}=\frac{{\mathrm{AUC}}_{\mathrm{extravenous}}\times I.V.\;\mathrm{dose}}{{\mathrm{AUC}}_{I.V.}\times \mathrm{extravenous}\;\mathrm{dose}}\;$$

#### 2.10.3. Histopathological Study of Rat Skin after Topical Administration of the Selected LNC Formulation

Twenty-four hours after the transdermal treatment, six rats were euthanatized to examine them for skin irritation. Specimens were stored in a 10% formalin solution of PBS with pH 7.4. Dehydrated specimens were embedded in paraffin. Each specimen was sliced into 5 µm cross-sections and dyed with hematoxylin. These samples were compared to healthy control skin under a microscope (A). Each skin sample was inspected for inflammatory cells or evidence of irritation in the different skin layers.

### 2.11. Statistical Analysis

Average values were computed for all tests, which were run in triplicate. Analysis of variance (ANOVA) and paired *t*-tests were employed in GraphPad^®^ Prism version 8.0.2 for statistical analysis; *p-* values less than 0.05 were regarded as significant.

## 3. Results and Discussion

### 3.1. Preparation and Characterization of Blank LNCs

The effects of using different oils, different surfactants, and different oil:surfactant ratios on the preparation of LNCs and their physical properties were investigated using 36 different formulations. Detailed results of the particle size, polydispersity index and zeta potential of the blank LNCs are shown in Table 1.

#### 3.1.1. Particle Size and PDI

The four types of oils used in the preparation of the LNCs showed a significant effect on the particle size of the obtained vesicles when using the same surfactant type and the same oil:surfactant ratio. Labrafil^®^ M1944CS and lavender oil produced LNCs with smaller particles than Labrafac™ PG and Miglyol^®^ 812. In formulations F4, F10, F1 and F7, with the same surfactant type (Kolliphor^®^ HS15) and oil:SAA ratio (1:1), the particle sizes of the LNCs were 32.26, 37.24, 64.95 and 74.5 nm for Labrafil^®^ M1944CS, lavender oil, Labrafac™ PG, and Miglyol^®^ 812, respectively. Labrafil^®^ M1944CS is a PEGylated medium-chain triglyceride that contains PEG moieties with significant surfactant properties, which led to a further decrease in the interfacial tension between the oil droplets and water, leading to the production of smaller LNCs. On the other hand, Labrafac and Miglyol are PEG-free triglycerides; hence, they lack the emulsifying effect and act only as oily cores. In the case of lavender oil, it is an essential oil with low viscosity and low oil-to-water interfacial tension. Therefore, size reduction is easier to achieve, as lower energy is required. Moreover, the polarity of lavender oil makes it a suitable candidate for the formation of nanoemulsions through the phase-inversion method [27]. Both of these factors explain the small particle sizes obtained for the lavender-oil-based LNCs.

**Figure 2:**
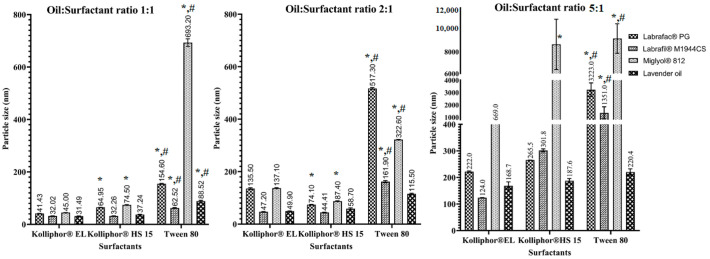
The effect of surfactant type and oil:surfactant ratio on the particle size of LNCs. All values are presented as the mean ± SD (*n* = 3). * Significant difference in comparison to Kolliphor^®^ EL. ^#^ Significant difference in comparison to Kolliphor^®^ HS.

Regarding the types of surfactants used, there was a tendency to produce smaller nanovesicles when using Kolliphor^®^ EL and Kolliphor^®^ HS15 compared to Tween 80 (Figure 2), which was consistent with the findings of [28]. This could be related to the composition of the surfactants, as Kolliphor^®^ EL and Kolliphor^®^ HS15 are high-molecular-weight PEGylated macrogol surfactants. They impart more stabilization to the oil droplets by steric hindrance due to their long fatty acid chains, which prevent oil aggregation. As a result, smaller and more homogeneous LNCs were produced using these two surfactants compared to Tween 80.

It should be noted that the phase-inversion temperature was the highest in the case of Tween 80, followed by Kolliphor^®^ EL, and the lowest in the case of Kolliphor^®^ HS15. This indicates that Kolliphor^®^ HS15 requires less heating time to achieve the phase inversion, thereby reducing the exposure of the loaded drugs to high temperatures.

LNCs were prepared in three increasing oil:surfactant ratios; the smallest particle size values were associated with LNCs prepared using the oil:surfactant ratio of 1:1 (Figure 2). This finding can be attributed to the ability of the surfactant added to reduce the interfacial tension between the oil droplets and the aqueous phase, thereby producing smaller LNCs [29]. At higher oil:surfactant ratios, the particle size was increased due to the decrease in the emulsifying power of the surfactant at higher oil contents, and particle size reduction became more difficult to achieve. Formulations with a high surfactant content displayed low polydispersity index values, indicating the homogeneity of the prepared nanocarriers, since higher surfactant concentrations imparted more stabilization to the LNCs and prevented oil agglomeration. LNCs prepared using oil:surfactant ratios of 1:1 and 2:1 were highly stable, as demonstrated by their small particle sizes, narrow PDI indices, and negatively charged surfaces. Meanwhile, the oil:surfactant ratio of 5:1 resulted in the production of very large droplets (above 200 nm) that were not considered as LNCs.

#### 3.1.2. Zeta Potential

The zeta potential of the prepared LNCs was found to be negative, ranging from −10 to −36 mV, which offers sufficient static stabilization of the particles. The negative charge of the LNCs could be attributed to the incorporation of lecithin and the acidic nature of the oils used in the preparation of the LNCs. Lecithin is an ampholytic surfactant that contains phosphate and amino groups. The negative values of the zeta potential indicated that the phosphate groups predominated the charge of the LNCs, as they were thought to be directed towards the external aqueous phase [30]. Nevertheless, the acidic nature of the oily core justifies the negative potential of the particles, as reported in [24].

LNCs are colloidal systems that are stabilized using high-molecular-weight surfactants (macrogols) that exert steric effects and restrict the mobility of the particles, preventing them from aggregation [31,32]. Therefore, the negatively charged surfaces of LNCs, along with their low PDI values and the steric effects of the macrogol surfactants used in their preparation, were all indications of the excellent stability of the nanocapsules [30].

On the other hand, zeta potential was observed to become more negative by increasing the oil:surfactant ratio, which can be attributed to the acidic nature of the oils [24]. In addition, low surfactant concentrations are thought to increase the mobility of the nanoparticles and, hence, increase the zeta potential. However, no linear correlation was observed between the types of either oils or surfactants and the zeta potential of the LNCs.

### 3.2. Preparation and Characterization of Asenapine-Maleate-Loaded LNCs

**Figure 3:**
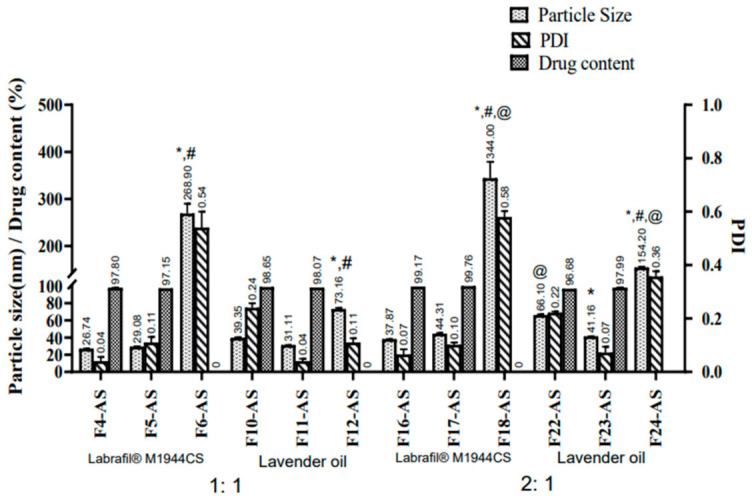
The physical characterization of ASP-loaded LNCs. All values are presented as the mean ± SD (*n* = 3). * Significant difference in comparison to the formulation containing Kolliphor^®^ HS with the same oil type and ratio. ^#^ Significant difference in comparison to the formulation containing Kolliphor^®^ EL with the same oil type and ratio. ^@^ Significant difference in comparison to the formulation with an oil:surfactant ratio of 1:1 with the same oil and surfactant type.

From the above results, it could be concluded that the use of Labrafil^®^ M1944CS and lavender oil as oily cores with oil:surfactant ratios of 1:1 and 2:1 were optimal for the delivery of asenapine in terms of particle size, PDI and zeta potential. Therefore, they were selected for further studies, which included ASP encapsulation. The three different surfactants used previously were again investigated to screen their ability to aid in the solubilization of the entrapped drug. The physical characterization of 12 ASP-loaded LNCs is shown in Figure 3.

#### 3.2.1. Particle Size and PDI

The effect of drug loading on the particle size was manifested in the reduced mean vesicle diameter of the ASP-loaded LNCs compared to their corresponding blank formulations. This could be attributed to the interaction between the drug and the hydrophobic regions of the surfactants, which led to better packing and reduction in the mean vesicle diameter [33].

As already noted in the blank formulations, loaded LNCs with a lower oil:surfactant ratio (1:1) yielded smaller particles than the other ratio (2:1). However, both ratios produced LNCs with very small particles (ranging between 26 and 66 nm) and low PDI values (ranging from 0.04 to 0.2), except for the LNCs prepared with Tween 80. Drug-loaded LNCs prepared using Tween 80 displayed larger diameters at high and low surfactant concentrations with both Labrafil^®^ M1944CS (268.9 and 344 nm) and lavender oil (73.16 and 154.2 nm). The instability of Tween-80-based LNCs was manifested in their large particle size and very broad PDI values; consequently, creaming and separation occurred upon standing.

#### 3.2.2. Zeta Potential

A significant difference in zeta potential was observed between the blank and loaded lipid nanocapsules. The blank LNCs were found to be more negative compared to the drug-loaded LNCs. ASP has a cationic moiety, so encapsulation of the drug resulted in a positive shift in the zeta potential.

#### 3.2.3. Drug Content

The drug content approached 100% in all of the drug-loaded LNCs. Tween 80-based LNCs that suffered from phase separation upon standing—namely, F6-AS, F12-AS, F18-AS and F24-AS—were excluded from this experiment. The observed full asenapine content confirmed the complete solubilization of the drug in the oily vehicle using very small volumes of methanol that was evaporated during the heating/cooling cycles. The solubility of asenapine was also confirmed under a light microscope that showed no existence of any drug crystals.

From the previous results, it was decided to exclude all of the Tween 80-containing formulations from the next investigations, and to proceed to the skin permeation experiments only with formulations containing Kolliphor^®^ EL and Kolliphor^®^ HS 15.

### 3.3. Ex Vivo Skin Permeation and Deposition Study

**Figure 4:**
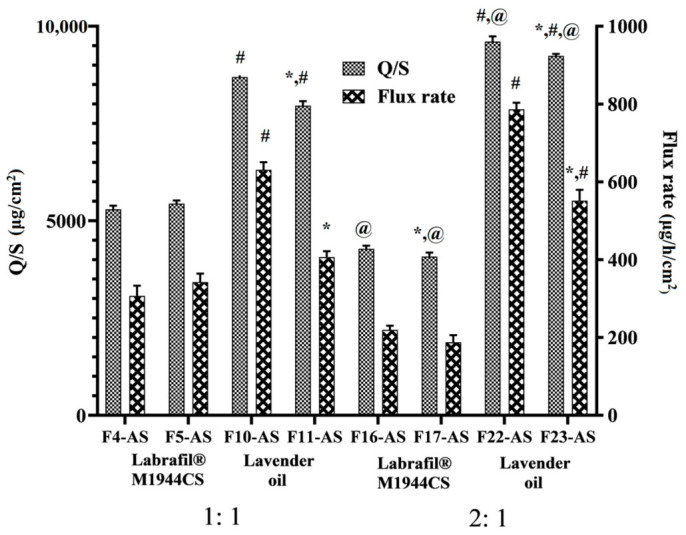
The amounts of asenapine permeated per surface area and flux rates of ASP from the different LNCs. All values are presented as the mean ± SD (*n* = 6). * Significant difference in comparison to the formulation containing Kolliphor^®^ HS with the same oil type and ratio. ^#^ Significant difference in comparison to the formulation containing Labrafil^®^ M1944CS with the same surfactant type and oil ratio. ^@^ Significant difference in comparison to the formulation with an oil:surfactant ratio of 1:1 with the same oil and surfactant type.

This experiment was carried out to evaluate the ability of LNCs to boost the permeability of ASP across excised rat skin. The type of oil greatly affected the permeation of the ASP. The results presented in Figure 4 revealed that the permeation of asenapine was significantly increased with LO-LNCs (79–96%) compared to Labrafil^®^-based formulations (42–54%). Higher flux rates and amounts permeated through the skin were observed for F10-AS, F11-AS, F22-AS and F23-AS than for F4-AS, F5-AS, F16-AS and F17-AS.

These results could be explained by the fact that lavender oil is an essential oil containing high contents of terpenes that fluidize the stratum corneum and disrupt its tight packing [13]. Therefore, lavender oil served simultaneously as an oily vehicle and a permeation enhancer, while Labrafil^®^ M1944CS acted only as a vehicle for the drug.

The LO-LNCs exhibited surfactant-concentration-independent behavior, since the permeation effect of the oil surpassed that of the surfactant. The same observation was reported by [15], and it was explained that the release of ibuprofen from the LNCs was prompted by the lipid matrix itself rather than the surfactants. Meanwhile, with Labrafil^®^-based LNCs, the surfactant is the only ingredient that boosts the permeation across the skin. Therefore, the effect of the oil:surfactant ratio differed according to the type of oil. In LO-LNCs, the increase in the oil:surfactant ratio led to increased permeation of the drug across the rat skin. This could be attributed to the interaction of lavender oil with the outer skin layer and the exertion of its significant permeation enhancement effect, as explained above. F22 with an oil:surfactant ratio of 2:1 displayed the highest permeation—approximately 96% of the applied dose was permeated.

On the other hand, the surfactant concentration governed the permeation of ASP delivered by Labrafil^®^-based LNCs, since the surfactant increases the fluidization of the stratum corneum, solubilizes the drug, and increases its partitioning through deep skin layers. As the oil:surfactant ratio increased, the permeation of the drug decreased due to the decreased emulsifying power of the surfactant. The cumulative permeation of the ASP was found to be increased in the case of the lower oil:surfactant ratio (1:1) compared to the higher ratio (2:1) for both Kolliphor^®^ HS 15 (5292.74 and 4281.31 µg/cm^2^, respectively) and Kolliphor^®^ EL (5292.74 and 4081.51 µg/cm^2^, respectively).

In terms of the effect of the surfactant type on the permeation process, Kolliphor^®^ HS 15 was observed to increase the permeation compared to Kolliphor^®^ EL in all formulations, with only one exception (F4). This observation favored the use of Kolliphor HS15 in the in vivo studies.

**Figure 5:**
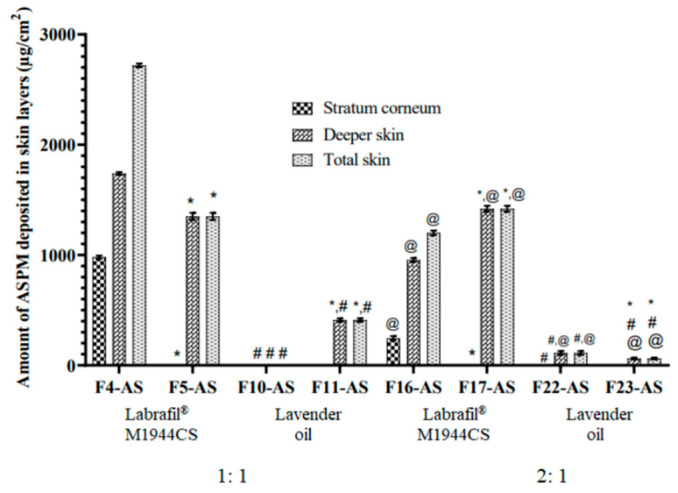
Deposition of asenapine from ASP-loaded LNCs in different skin layers. All values are presented as the mean ± SD (*n* = 6). * Significant difference in comparison to the formulation containing Kolliphor^®^ HS with the same oil type and ratio. ^#^ Significant difference in comparison to the formulation containing Labrafil^®^ M1944CS with the same surfactant type and oil ratio. ^@^ Significant difference in comparison to the formulation with an oil:surfactant ratio of 1:1 with the same oil and surfactant type.

The skin deposition results were consistent with the permeation results. In LO-LNCs, minimal amounts of asenapine (if any) were detected in the skin layers, since almost all of the applied dose crossed the skin and was detected in the receptor compartment. On the other hand, higher amounts of the drug were deposited in both the stratum corneum and the deeper skin layers with Labrafil-based LNCs, as shown in Figure 5.

The results of skin permeation and deposition revealed that drug permeation from the LNCs was dependent on the lipid carrier–skin interactions. Most probably, the LNCs disintegrated on the skin surface, and their components—along with the drug—became accessible to the skin, facilitating their permeation across the SC. Nevertheless, the hydrating effect of these oils also plays a great role in increasing skin hydration, resulting in swelling and fluidization of the SC, with subsequent higher drug permeation [34].

Based on the aforementioned results, the formulation F22-AS, containing lavender oil and Kolliphor^®^ HS15 at a ratio of 2:1, displayed the best physical features and the highest ASP permeability through rat skin. Consequently, it was decided to move further with additional research using this formulation.

### 3.4. Evaluation of the Physical Stability of the Selected ASP-Loaded LNCs upon Storage

The optimized formulation (F22-AS) was stored for six months to evaluate its physical stability. The particle size and size homogeneity in terms of PDI for both the fresh and stored formulations for two months and six months were compared (Table 2). Minor changes were observed in the particle size of the stored formulations, which indicated the stability of the loaded LNCs over 6 months, despite being statistically different. The PDI and zeta potential values did not show significant differences throughout the storage of the LNCs. F22-AS showed excellent stability in terms of particle size, PDI, and zeta potential over six months.

### 3.5. High-Resolution Transmission Electron Microscopy (HR-TEM)

**Figure 6:**
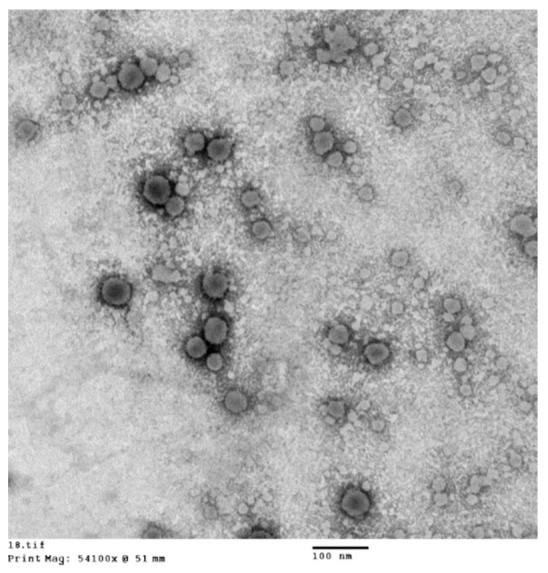
Visualization of ASP lipid nanocapsules (F22-AS) showing dense shells and light cores.

The electron micrographs of formulation F22-AS showed round-shaped particles with clear cores and dense external shell morphology, as previously reported by [12,35]. The particle size analysis results were consistent with the apparent particle sizes in the TEM micrographs (Figure 6).

### 3.6. Fourier-Transform Infrared (FTIR) Spectroscopy

**Figure 7:**
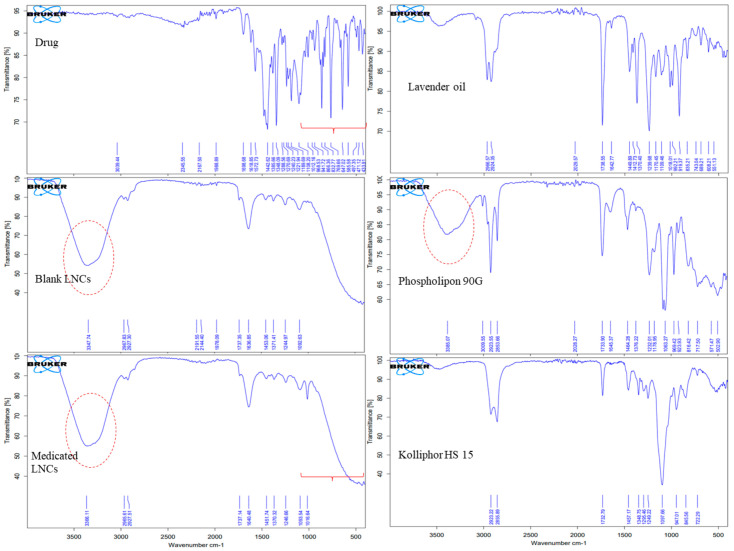
FTIR spectra of the drug, excipients, and blank and loaded LNCs (F22-AS).

FTIR spectroscopy was used to examine the drug’s interaction with all of the components used to prepare the LNCs. Figure 7 depicts the FTIR spectra of asenapine, lavender oil, Kolliphor^®^ HS 15, Phospholipon 90 G, blank LNCs and ASP LO-LNCs.

Asenapine’s spectrum displayed distinctive bands at 3039 cm^−1^ (representing aromatic C–H) 1698 cm^−1^ (for C=O stretching), 1572 cm^−1^ (C=C stretching vibration), 1442 cm^−1^ (C–C stretching), 1348 cm^−1^ (C–N stretching), 1189 cm^−1^ (C–O stretching), and 760 cm^−1^ (C–O stretching and C–Cl stretching) [36].

The characteristic bands observed from the IR spectra of Kolliphor^®^ HS15 included a shallow peak at approximately 3400 cm^−1^ due to hydrogen bonding, stretching modes of =C–H and C–H between 3200 and 2660 cm^−1^, an ester group (C=O stretching) at 1732 cm^−1^, and a strong peak of C–O stretching at 1097 cm^−1^. The IR spectra of Kolliphor^®^ HS15 were consistent with the results reported by [23,37].

The IR spectra of lavender oil were found be to identical to those discussed by [38,39]. The band that appeared at 3400 cm^−1^ was attributed to O–H stretching vibrations of the hydrogen bond. The IR peaks in the spectral region of 3000–2800 cm^−1^—specifically at 2966, 2924 and 2869 cm^−1^—belonged to –CH2 stretching. The very intense IR peak shown at 1738 cm^−1^ was due to C=O stretching vibrations of esters. C=C stretching vibration appeared at 1642cm^−1^. The spectral region below 1500 cm^−1^ showed several vibration modes, including bands identified at 1370, 1412 and 1449 cm^−1^ (C–H bending vibrations), 1109, 1170 and 1239 cm^−1^ (C–O stretching), 992 cm^−1^ (–CH2 vibrations) and finally, strong bands at 835 and 919 cm^−1^ (C–H vibrations).

The FTIR spectrum of Phospholipon 90 G (PL 90 G) displayed similar bands to those reported by Beg et al. in 2016 [40]. The stretching peak of O–H appeared at 3400 cm^−1^, while that of –CH2 vibrations appeared in the 3000–2800 cm^−1^ region. An aliphatic =C–H peak was noticed at 3009 cm^−1^. The intense peak of C=O was found at 1733 cm^−1^. The vibrations of C=C, P=O, and C–O stretching appeared at 1645 cm^−1^, 1376 cm^−1^ and 1232 cm^−1^, respectively, along with a P–C band and P–O vibrations at 1178 and 1063 cm^−1^, respectively [40].

The IR spectrum of the blank LO-LNCs formulation showed the characteristic peaks of the included ingredients—Kolliphor^®^ HS15 and Phospholipon 90G. This denotes the interaction of the components to generate a homogenous blend representing the nanocapsules’ shells. The peaks of ASP disappeared in the case of ASP-loaded LO-LNCs, indicating complete encapsulation of the drug inside the vesicle core. Moreover, the quite broad O–H peak appeared as a single peak, while the quaternary nitrogen peak of the drug was relatively suppressed, confirming the merging of those two functional peaks and the interaction of the drug with the LNCs.

In conclusion, the FTIR study of the formulation F22-AS confirmed the interaction of the surfactant and lecithin to form the outer shell, confirming the successful preparation of blank lipid nanocapsules. In addition, asenapine was completely loaded in the oily core and encapsulated by the surfactant shell of the nanocapsules.

### 3.7. Bioavailability Study

#### 3.7.1. In Vivo Pharmacokinetic Analysis

**Figure 8:**
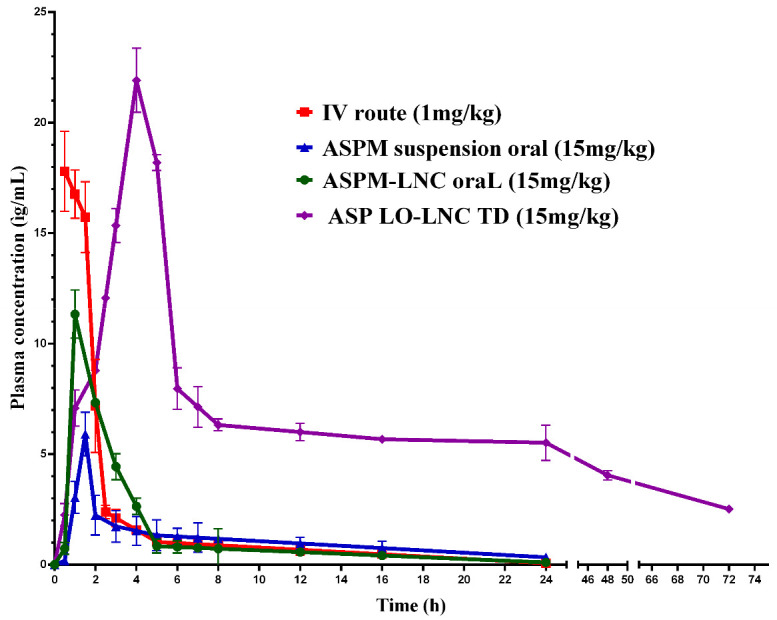
Plasma concentrations of asenapine after IV, oral and transdermal administration.

F22-AS was selected for the in vivo pharmacokinetic study to be assessed as a potential carrier for the transdermal delivery of asenapine. For comparison, ASP was also administered through the IV and oral routes in solution and suspension forms, respectively. In order to test the LNCs as promising oral drug delivery carriers that enhance the bioavailability of the drug, F22-AS was also administered orally using the same dose used for the transdermal administration. Plasma concentrations of asenapine were plotted against time, as shown in Figure 8, and the pharmacokinetic parameters of each route are listed in Table 3.

The study revealed that the ASP-loaded lipid nanocapsules enhanced the oral absorption of asenapine, as indicated by its higher C_max_ value of 11.33 μg/mL compared to the C_max_ value of the drug suspension (5.91 μg/mL). However, the asenapine plasma concentrations for both dropped rapidly due to the hepatic metabolism, which is considered to be the major obstacle to the oral administration of asenapine. Consequently, the oral bioavailability of both the drug suspension and loaded LNCs was very low (3.75 and 4.45%, respectively).

On another note, the AUC_0–72_ value for the transdermal route was found to be 372.10 μg × h/mL, while the AUC_0–24_ values of the oral drug suspension and loaded LNCs were found to be 25.59 and 31.88 μg × h/mL, respectively. The extent of absorption, as indicated by the AUC values, was significantly greater (*p* < 0.05) with the transdermal route than with oral administration. The absolute bioavailability of the transdermal delivery of asenapine from the LNCs was enhanced greatly (up to 52%). This significant increase in bioavailability following the transdermal application of the lipidic carriers was due to the avoidance of the extensive hepatic metabolism associated with oral administration. The transdermal lipid nanocapsules achieved a maximum drug plasma concentration (C_max_) of 21.9 µg/mL, whereas the C_max_ of the oral asenapine suspension and ASP-loaded LNCs was 5.9 µg/mL and 11.3 µg/mL, respectively. In addition, the elimination half-life (t_1/2_) and MRT values were also significantly greater (*p* < 0.05) for transdermal than oral administration, indicating the delayed absorption and slow elimination from the transdermal route. This could have been due to the continuous and sustained delivery of the drug from the transdermal formulation. The slow release of the drug from lipid nanocapsules and penetration through the skin barrier into the blood resulted in a sustained release profile over 72 h.

Deposition of asenapine in rat skin was also determined after 24 h; the deposition of asenapine following transdermal application of lipid nanocapsules was 1.165 μg/cm^2^ ± 0.40, which represented only 0.5% of the applied dose.

#### 3.7.2. Lipid Nanocapsules Versus Invasomes for Transdermal Drug Delivery

Previous works have studied the transdermal delivery of ASP via invasomes. Invasomes are soft vesicular nanosystems that are prepared using natural terpenes as penetration enhancers for enhancing drug permeation through the skin [41]. The pharmacokinetics of two types of lipidic nanoparticles—nanocapsules and nanovesicles—were compared. Lipid nanocapsules were found to achieve higher C_max_ (21.9 μg/mL) than invasomes (9.4 μg/mL) upon transdermal application. The increased and rapid permeation of asenapine from LNCs could be related to the fact that the LNCs dissolved on the outer skin, releasing their ingredients. Lavender oil and nonionic surfactants interact with the SC and penetrate the intercellular regions, leading to increased fluidity, solubility and extraction of lipid components combined with the disruption of the corneocytes, with subsequently increased permeation [34]. Meanwhile, in the case of invasomes, the drug is entrapped in the lipid bilayer membrane of the vesicles, leading to slow release and a lower flux rate. This hypothesis was augmented by the in vivo skin deposition results, as greater amounts of asenapine were detected in the skin treated with invasomes (5.22 μg/cm^2^) than in that treated with LNCs (1.16 μg/cm^2^). Invasomes were found to accumulate in the skin layers, forming a drug reservoir that led to a sustained release of asenapine.

**Figure 9:**
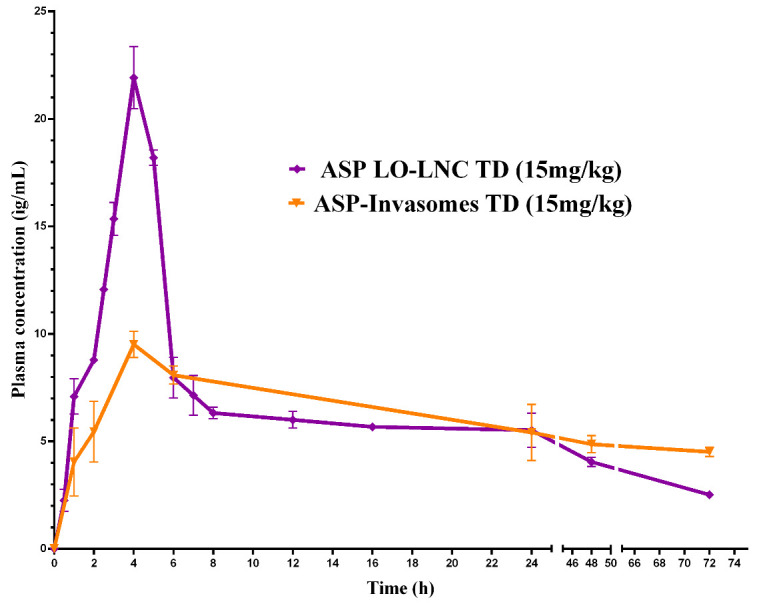
In vivo plasma profile of asenapine-loaded lipid nanocapsules (F22-AS) and invasomes (F2-AS) for transdermal drug delivery.

Both of the developed formulations showed excellent sustained release profiles and achieved comparable BAV values that could maximize the therapeutic outcomes in the pharmacological treatment of schizophrenia. However, invasomes had the additional advantage of a more stable drug plasma concentration of asenapine maleate after single-dose administration, as shown in Figure 9. This could consequently minimize the annoying side effects of second-generation antipsychotics, which are considered to be among the main reasons for medication nonadherence.

#### 3.7.3. Histological Evaluation

**Figure 10:**
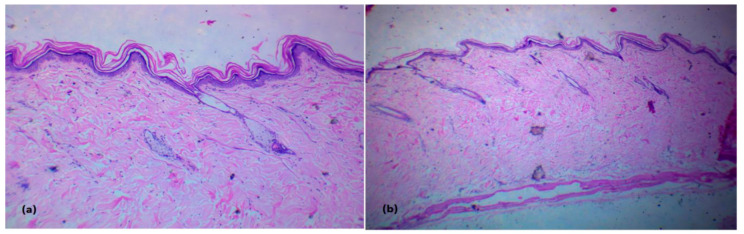
H&E sections: (**a**) normal skin; (**b**) skin treated with LO-LNCs with no signs of inflammation.

The histopathology of the skin was studied by hematoxylin/eosin staining. Histopathological microscope images of untreated skin and skin treated with LO-LNCs are displayed in Figure 10. The light micrographs showed no infiltration of inflammatory cells in different layers of the skin, indicating the safety of the examined formulation for topical application without the potential for irritation. Comparable histological results were reported for the use of lipid nanocapsules for the transdermal delivery of ropivacaine [42].

## 4. Conclusions

This study emphasizes the fruitful use of essential oil (Lavender oil) as a novel oily phase compared to MCT oily phases—namely, Labrafil^®^ M1944CS and Labrafac™ PG—for the preparation of lipid nanocapsules as a very promising transdermal delivery system for drugs (especially hydrophobic ones). The high contents of terpenes in lavender oil are the primary root of its significant permeation enhancement effect, due to the interaction with the outer skin layer and fluidization of the stratum corneum, disrupting its tight packing. The optimized LNC formulation displayed a relatively small particle size (66.1 ± 0.8711) and significant ex vivo permeation of asenapine, along with optimal stability during storage for six months. Sustained transdermal delivery of ASP for up to 72 h was achieved, which could reduce the dosing frequency, enhance bioavailability, and improve patient compliance.

## Figures and Tables

**Table 1:** Composition of the different blank LNC formulations.

Formulation Code	Oil Type	Surfactant Type	Oil: Surfactant Ratio	Particle Size ± S.D (nm)	PDI ± S.D	Zeta Potential ± S.D (mV)
F1	Labrafac™ PG	Kolliphor^®^ S15	1:1	64.95 ± 0.19	0.233 ± 0.003	−13.8 ± 0.6
F2		Kolliphor^®^ EL	1:1	41.43 ± 0.23	0.077 ± 0.017	−12.6 ± 1.27
F3		Tween 80	1:1	154.6 ± 1.75	0.278 ± 0.006	−10.2 ± 0.551
F4	Labrafil^®^ M1944CS	Kolliphor^®^ HS15	1:1	32.26 ± 0.43	0.259 ± 0.005	−13 ± 0.781
F5		Kolliphor^®^ EL	1:1	32.02 ± 0.18	0.099 ± 0.012	−9.53 ± 0.583
F6		Tween 80	1:1	62.52 ± 1.29	0.238 ± 0.01	−13.8 ± 0.985
F7	Miglyol^®^ 812	Kolliphor^®^ HS15	1:1	74.5 ± 0.77	0.211 ± 0.012	−18 ± 1.02
F8		Kolliphor^®^ EL	1:1	45 ± 0.08	0.174 ± 0.007	−10.8 ± 0.361
F9		Tween 80	1:1	693.2 ± 14.19 *	0.212 ± 0.019	−33 ± 0.854
F10	Lavender oil	Kolliphor^®^ HS15	1:1	37.24 ± 1.2	0.156 ± 0.034	−9.8 ± 0.8
F11		Kolliphor^®^ EL	1:1	31.49 ± 0.43	0.166 ± 0.023	−19.4 ± 1.04
F12		Tween80	1:1	88.52 ± 3.23	0.317 ± 0.013	−13 ± 0.4
F13	Labrafac™PG	Kolliphor^®^ HS15	2:1	74.1 ± 1.021	0.168 ± 0.017	−27.5 ± 2.65
F14		Kolliphor^®^ EL	2:1	135.5 ± 2.93	0.209 ± 0.006	−15.7 ± 0.954
F15		Tween 80	2:1	517.3 ± 2.93 *	0.199 ± 0.006	−23 ± 2.53
F16	Labrafil^®^ M1944CS	Kolliphor^®^ HS15	2:1	44.41 ± 1.103	0.24 ± 0.008	−20.2 ± 1.08
F17		Kolliphor^®^ EL	2:1	47.2 ± 0.85	0.228 ± 0.005	−11.8 ± 0.289
F18		Tween 80	2:1	161.9 ± 3.9	0.319 ± 0.042	−23.4 ± 0.416
F19	Miglyol^®^ 812	Kolliphor^®^ HS15	2:1	87.4 ± 1.336	0.098 ± 0.014	−27 ± 1.46
F20		Kolliphor^®^ EL	2:1	137.1 ± 1.94	0.268 ± 0.002	−18.5 ± 0.379
F21		Tween 80	2:1	322.6 ± 0.05 *	0.447 ± 0.015	−36.8 ± 1.97
F22	Lavender oil	Kolliphor^®^ HS15	2:1	104.2 ± 0.05	0.584 ± 0.011	−20.9 ± 0.458
F23		Kolliphor^®^ EL	2:1	69.63 ± 0.5	0.157 ± 0.021	−18.3 ± 1
F24		Tween 80	2:1	115.5 ± 2.34	0.181 ± 0.009	−18.4 ± 0.404
F25	Labrafac™ PG	Kolliphor^®^ HS15	5:1	265.5 ± 0.075 *	0.225 ± 0.011	−23.5 ± 2.14
F26		Kolliphor^®^ EL	5:1	222 ± 2.6 *	0.217 ± 0.016	−21.6 ± 2.86
F27		Tween 80	5:1	3223 ± 552 *	1.000 ± 0.000	−30 ± 2.15
F28	Labrafil^®^ M1944CS	Kolliphor^®^ HS15	5:1	301.8 ± 5.6 *	0.369 ± 0.011	−31.4 ± 0.79
F29		Kolliphor^®^ EL	5:1	124 ± 0.5	0.214 ± 0.003	−34.1 ± 0.45
F30		Tween 80	5:1	1351 ± 491 *	1.000 ± 0.000	−23 ± 0.173
F31	Miglyol^®^ 812	Kolliphor^®^ HS15	5:1	8671 ± 2293 *	0.363 ± 0.331	−39.6 ± 33.3
F32		Kolliphor^®^ EL	5:1	669 ± 13 *	0.293 ± 0.027	−27.8 ± 1.63
F33		Tween 80	5:1	9201 ± 1345	0.437 ± 0.025	−45.08 ± 0.713
F34	Lavender oil	Kolliphor^®^ HS15	5:1	187.6 ± 8.1	0.259 ± 0.24	−26.7 ± 0.55
F35		Kolliphor^®^ EL	5:1	168.7 ± 14.6	0.377 ± 0.039	−34 ± 1.63
F36		Tween 80	5:1	220.4 ± 12.08 *	0.217 ± 0.009	−26.9 ± 2.06

Phospholipon^®^ 90G and NaCl were kept constant in all formulations at concentrations of 1% *w*/*w*. * Formulations with a mean particle size of more than 200 nm were excluded from the study.

**Table 2:** The effects of storage of the selected ASP-loaded LNCs (F22-AS) on particle size, PDI and zeta potential.

	Particle Size (nm)	P.D.I	Zeta Potential (mV)
Fresh	66.1 ± 0.87	0.22 ± 0.005	−5.16 ± 0.13
2 months	68.94 *± 0.75	0.244 ± 0.07	−4.61 ± 0.34
6 months	71.4 * ± 0.23	0.257 ± 0.024	−5.01 ± 0.34

All values are presented as the mean ± SD (*n* = 3). * Significant difference in comparison to fresh LNCs.

**Table 3:** In vivo pharmacokinetic parameters of transdermal application of optimized ASP-loaded LNCs compared to intravenous and oral routes.

Route of Administration		Intravenous	Oral		Transdermal
Parameter	Unit	ASP solution	ASP suspension	ASP-loaded LNCs F22-AS	ASP-loaded LNCs F22-AS
Dose	mg/kg	1 ± 0	15 ± 0	15 ± 0	15 ± 0
T_max_	h	0	1.5 ± 0	1 ± 0	4 ± 0
C_max_	μg/mL	18.89 ± 1.28	5.91 ± 0.481	11.33 ± 2.03	21.91 ± 1.61 *
AUC 0–t last	μg × h/mL	47.68 ± 2.76	25.59 ± 1.48	31.88 ± 1.95	372.10 ± 26.41 *^,#^
AUC 0–∞	μg × h/mL	48.00 ± 2.78	29.35 ± 2.08	32.59 ± 4.03	525.77 ± 21.34 *^,#^
MRT 0–∞	h	3.52 ± 0.25	11.95 ± 0.56	5.33 ± 0.32	58.73 ± 4.32 *^,#^
Bioavailability	%	-	3.57 ± 0.27	4.45 ± 0.96	52.02 ± 6.01 *^,#^
Skin deposition	μg/cm^2^	-	-	-	1.165 ± 0.40

All values are presented as the mean ± SD (*n* = 6). * Significant difference in comparison to ASP suspension (oral). ^#^ Significant difference in comparison to ASP-loaded LNCs (oral).

## Data Availability

Not applicable.
